# Scoliosis Surgery in Cystic Fibrosis: Surgical Considerations and the Multidisciplinary Approach of a Rare Case

**DOI:** 10.1155/2016/7186258

**Published:** 2016-06-19

**Authors:** George I. Mataliotakis, Athanasios I. Tsirikos, Karen Pearson, Don S. Urquhart, Carolyn Smith, Andrew Fall

**Affiliations:** Scottish National Spine Deformity Centre, Royal Hospital for Sick Children, Sciennes Road, Edinburgh EH9 1LF, UK

## Abstract

Spinal deformity in patients with cystic fibrosis (CF) is usually mild requiring no treatment. These patients are rarely considered as surgical candidates for scoliosis correction, as the pulmonary condition and other comorbidities increase the risk of general anaesthesia and recovery. This paper reviews all the literature up to date with regard to scoliosis in patients with CF and reports this unique case of a 14-year-old Caucasian girl with progressive scoliosis, who was treated surgically at the age of 17. She underwent a posterior spinal fusion T2-L3 with the use of unilateral segmental instrumentation. Preoperative workup included respiratory, cardiac, anaesthetic, endocrine, and dietician reviews, as well as bone density optimisation with zoledronic acid and prophylactic antibiotics. Surgical time was 150 minutes and intraoperative blood loss was 47% of total blood volume. Postoperative intensive care included noninvasive ventilation, antibiotic cover, pain management, chest physiotherapy, pancreatic enzyme supplementation, and nutritional support. She was discharged on day 9. At follow-up she had a good cosmetic outcome, no complaints of her back, and stable respiratory function. Multidisciplinary perioperative care and meticulous surgical technique may reduce the associated risks of major surgery in CF patients, while achieving adequate deformity correction and a good functional outcome.

## 1. Introduction

Cystic fibrosis (CF) is the most common life-limiting autosomal recessive disease in the United Kingdom, affecting over 10,000 children and young adults. Average life expectancy is currently 41 years [1]. Chronic mucus hypersecretion leads to recurrent lower respiratory tract infections and lung disease, while nutrition and bone health may be adversely impacted upon due to exocrine pancreatic insufficiency, vitamin deficiencies, and inflammation.

A number of later-onset musculoskeletal manifestations of CF are reported including scoliosis, kyphosis, low bone mineral density, and pathological fractures [2–10].

Case series of children and adolescents with CF and scoliosis have been reported by a number of centres including our own centre [2–4, 7, 9, 10]. However, the paradigm of a radiologically significant (Cobb angle > 10°) versus a clinically important scoliosis remains a key message from these reports. Reduced respiratory reserve when present due to underlying lung disease may make CF patients unsuitable surgical candidates; however the spinal deformity is usually mild and does not require surgical correction [3, 7, 10].

We report a young woman with CF and progressive thoracic scoliosis, who underwent surgical treatment. To our knowledge, this is the first report of a CF patient who underwent posterior spinal fusion for scoliosis.

## 2. Case Report

### 2.1. History and Examination

A Caucasian girl with CF (genotype: F508del/F508del) was referred to clinic with a double thoracic scoliosis, at the age of 14.3 years. This was an incidental finding when a chest X-ray was obtained as part of regular respiratory reviews. On examination, she had a rib hump and small thoracic translocation to the right with associated waistline asymmetry and prominence of the left side of the pelvis. Her shoulders and pelvis were level. Neurological examination and spinal MRI were normal. Mild ligamentous laxity was detected. Spinal radiographs showed a left upper thoracic and right low thoracic curve measuring 23° and 36°, respectively. Sagittal balance was normal and the Risser grade was 0. She was 1.3 years after menarche with positive history of mild scoliosis in the maternal extended family, which never required treatment.

### 2.2. Treatment

An underarm brace was attempted with the aim to stop scoliosis progression but was poorly tolerated by the patient. Over 2.5 years of monitoring both curves gradually deteriorated and the patient developed persistent mechanical back pain. Scoliosis correction was offered and a detailed preoperative assessment (cardiac, respiratory, anaesthetic, endocrine, and dietician) was organised. The cardiac review showed no anomalies.

### 2.3. Respiratory

Lung function had been stable for many months prior to surgery (FEV-1: 1.66 litres/59% predicted; FVC: 2.25 litres/69% predicted) and oxygen saturation was 98% in air; no further lung plethysmography was performed. Her sputum was chronically infected with* Stenotrophomonas maltophilia* and Methicillin-Resistant* Staphylococcus aureus*.* Pseudomonas aeruginosa* had been previously isolated and she had also had treatment for allergic bronchopulmonary* Aspergillosis* (ABPA). She had undergone Nissen fundoplication aged 6 years due to severe gastroesophageal reflux. She had severe bilateral bronchiectasis demonstrable on CT scan and received regular courses of intravenous antibiotics, daily nebulised mucolytic (dornase-alfa) and antibiotic (alternating colistin/tobramycin) treatments. She had a portacath (Port-a-Cath*™*) implantable venous access device. In addition, she received nutritional supplements, pancreatic enzyme replacement therapy, and daily supplementation of calcium, as well as vitamins A, D, E, and K. Her body weight was 54 kg with normal BMI. She has had previous adverse reactions to antibiotics (vancomycin, ceftazidime, teicoplanin, piperacillin/tazobactam, ciprofloxacin, and levofloxacin) and received intravenous (IV) aztreonam and cotrimoxazole for 2 weeks before surgery.

### 2.4. Endocrine

Bone mineral density (BMD-lumbar spine DEXA scan) *Z* scores had been consistently <−2.5 since the age of 12. Zoledronic acid was administered IV 4 months prior to surgery in addition to her long-term calcium and vitamin D3 supplementation to improve bone strength.

### 2.5. Scoliosis Surgery

She underwent a posterior spinal fusion T2-L3 with the use of a single pedicle hook/screw/rod construct. Due to increased bleeding the side of the instrumentation was exposed first and the construct was finalised before the contralateral side was grafted with the use of autologous and allograft bone. Surgical time (skin incision to closure) was 150 minutes and blood loss 1700 mL (47% of total blood volume). The patient received transfusion of one unit of packed red cells, 260 mL of cell-salvaged blood, and 3 units of fresh frozen plasma (FFP) along with 1500 mL crystalloid fluid. Spinal cord monitoring (motor/sensory evoked potentials, EMG) was stable throughout the procedure.

### 2.6. Anaesthetic

Previous anaesthetics included laparoscopic fundoplication, portacath insertion, dental extractions, and numerous bronchoscopies. The portacath was utilised for induction of anaesthesia with initial propofol bolus of 120 mg followed by initiation of maintenance with total intravenous anaesthesia propofol and remifentanil target-controlled infusions and paralysis with atracurium. Her airway was easily managed and a size 6.5 mm reinforced endotracheal tube was sited with laryngoscopy direct grade 1 view. Several extra large-bore IV cannulae, a left radial arterial line, and a urinary catheter were then sited along with BIS forehead sensors for depth of anaesthesia monitoring. Active warming measures were employed and normothermia was achieved during procedure. Intraoperative analgesia included morphine, clonidine, and paracetamol. Initial tranexamic acid bolus of 2 grams was followed by an infusion of 500 mg/hour. Arterial systolic blood pressure was maintained between 85 and 100 mmHg throughout surgery with titration of GTN and remifentanil. The patient was successfully extubated at the end of the procedure, remained haemodynamically stable in recovery, and was transferred to PICU for ongoing care.

### 2.7. Postoperative Care

The patient was placed on noninvasive positive pressure ventilation. The pressures were weaned on day 2 postoperatively and the patient managed time off NIV on days 2 and 3, switching to nocturnal-only NIV support on day 3 with spontaneous ventilation on room air thereafter. She remained haemodynamically stable throughout her 4-day admission to PICU, alert, and interacting well with staff. She received a blood transfusion on day 3 in response to haemoglobin of 70 g/dL. Beclomethasone and salbutamol inhalers were restarted; antibiotic cover included aztreonam, cotrimoxazole, linezolid, and fluconazole as per microbiology recommendations. Pain management included IV morphine supplemented by clonidine, diazepam, paracetamol, and ibuprofen. This was stepped down to fentanyl patch and breakthrough sevredol on day 3. Pancreatic enzymes and vitamins were given as preoperatively along with supportive medication (ondansetron, cyclizine, omeprazole, macrogol, and picosulfate laxatives). She was transferred to the respiratory ward on day 4 after surgery.

**Figure 1:**
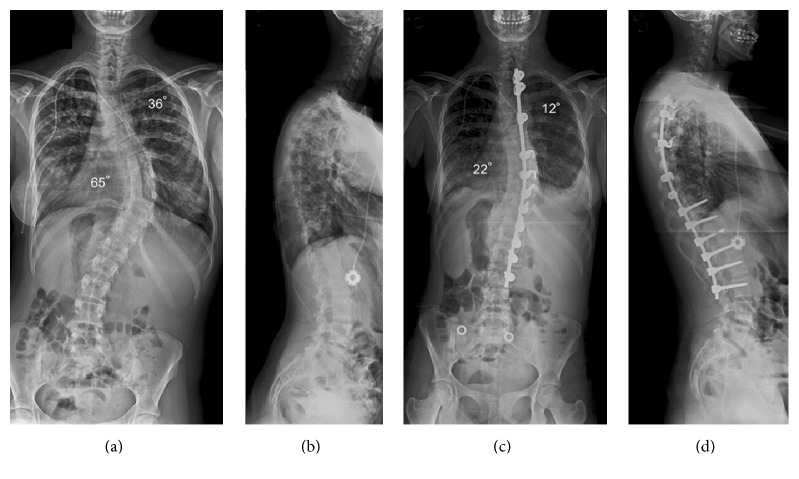
Preoperative posterior-anterior (a) and lateral (b) radiographs of the spine show a left upper and right low thoracic scoliosis measuring 36 and 65°, respectively. Posterior spinal fusion with the use of a right hook/screw and rod construct has achieved excellent correction of the curves to 12 and 22° and a satisfactory global coronal and sagittal balance for the spine (c, d).

Intensive chest physiotherapy and early mobilisation gradually improved right basal consolidation and the antibiotics were stopped on day 7 after surgery. Postoperative X-rays showed excellent correction of the double thoracic scoliosis and a balanced spine in both planes (Figure 1). She was fitted with a removable custom-moulded spinal jacket to wear when out of bed for 6 months in order to support the spine as the bone grafts were healing. She was discharged on the 9th postoperative day on paracetamol, tramadol, and gabapentin. Supplements were given beyond discharge until oral intake was optimised. At follow-up she had a well-healed scar, good spinal balance in the frontal and lateral planes, normal chest X-ray, and no respiratory or spinal complaints.

## 3. Discussion

The prevalence of scoliosis in CF ranges from 9.9 to 15.5%, has features of adolescent idiopathic scoliosis, and is greater in children older than 10 years (Table 1) [3, 7, 10]. This is higher than the 0.5–3.2% frequency of scoliosis recorded in the general population [4]. Common deformity pattern is that of nonprogressive thoracic curves with T6–T8 apex; scoliosis progression has been observed in 28.6% of adolescent patients with mean curves 12° and in 11.1% of adult patients despite the small curves of mean 15°, which is contrary to adolescent idiopathic scoliosis [3, 7]. This may be associated with the delayed bone age observed in patients with CF [10]. There is female gender predominance in the adolescent and adult groups (Table 1). Scoliosis size or side of the curve does not correlate with the severity of lung disease, bone age, or nutritional status [3, 10]. The presence of scoliosis is more common in patients with more severe lung disease [3].

No surgical treatment is usually required in CF patients with scoliosis due to the small curve size and low rate of progression resulting in mild deformity at skeletal maturity [4, 5, 7, 9, 11]. In contrast, a progressive thoracic scoliosis producing chest wall deformity as observed in our patient may further restrict lung function. A deformed chest may pose technical difficulties if lung transplant surgery was considered at a later age. In addition, our patient developed severe mechanical back pain which affected her activity level essential to preserve pulmonary function and quality of life.

A detailed preoperative assessment was critical in order to address the medical aspects of the condition and optimise the patient in view of major scoliosis surgery (Table 2). Lung function, risk of infection, bone density, and nutritional status were assessed preoperatively. The need for a multidisciplinary approach in the perioperative care of this group of patients cannot be overemphasized.

Medical comorbidities were addressed with optimisation of respiratory function through physiotherapy and prophylactic antibiotics. Bisphosphonates were introduced to improve BMD. Intraoperative blood loss was reduced using controlled hypotension and a meticulous surgical technique during exposure and instrumentation placement. Cell saver blood and products were given to accommodate anticipated blood loss. The single rod construct reduced surgical time, neurological risk, and risk of infection due to lesser implant density, achieved adequate deformity correction, and provided greater area for graft application to allow a solid fusion. At latest follow-up, our patient had an excellent cosmetic result, a balanced spine, and no respiratory complaints.

Scoliosis correction may be required in CF patients with progressive deformities which affect pulmonary function and produce pain. A meticulous preoperative assessment can reduce perioperative risks of major morbidity associated with the medical comorbidities of the condition. Posterior spinal fusion can achieve satisfactory deformity correction and a good functional outcome.

## Figures and Tables

**Table 1:** Previous studies presenting scoliosis data in patients with CF [3–8].

	Age groups	Mean age at diagnosis (range)	Number of patients	Male : female ratio	Scoliosis patients	Male : female ratio	Scoliosis angle (range)	Number of progressive curves/number of patients with scoliosis (percent, %)	Prevalence (%)
Erkkila et al. [4]	Infantile (0–4)	NR	58	NR	0	NR	—	NR	26^(*∗*)^
	4 to 16	NR	128	NR	7 (6%)	NR	NR (10–28)	NR	
	>19 (adult)	NR	17	NR	3 (18%)	NR	NR (16–26)	NR	12^(*∗∗*)^

Paling and Spasovsky-Chernick [9]	Infantile (0–4)	NR	32	NR	0	7 : 9	N/A	0	0
	4 to 16	15 (8–22)	151	96 : 55	15 (9.9%)	8 : 7	17 (10–31)	NR	9.93
	Adult (>16)						25 (15–31)	NR	

Kumar et al. [11]	0 to 4	2 (1–3)	42	1.8 : 1	5 (11.91%)	4 : 1	15 (10–16)	0	11.90
	4 to 16	9 (5–15)	90	1 : 1.6	14 (15.56%)	2 : 5	12 (11–25)	4/14 (28.57)	15.56
	Adult (16–43)	27 (17–43)	184	1.1 : 1	18 (9.78%)	2 : 5	15 (10–30)	2/18 (11.11)	9.78

Fainardi et al. [5]	0 to adult	10.9 (1.1–18)	319	1 : 1.1	7 (2.19%)	2 : 5	<20 (up to 37)	1/7 (14.29)	2.19

Fainardi and Bush [6]	10 to adult	(10–18)	173	NR	7 (4.05%)	NR	NR	NR	4.05

Hathorn et al. [7]	10 to adult	18 (10–18+)	143	NR	16 (11.19%)	NR	14 (10–38)	NR	11

^(*∗*)^Scoliosis and kyphosis in all age groups; ^(*∗∗*)^prevalence of scoliosis in ages from 15 to adult; N/A: not applicable; NR: not reported.

**Table 2:** Risks of scoliosis surgery in patients with CF and perioperative management.

	Perioperative risks	Action
Cystic fibrosis	Poor pulmonary reserve/restrictive lung disease	(1) Preoperative chest physiotherapy and increase in exercise
		(2) Noninvasive ventilation, chest physiotherapy, early mobilisation postoperatively
	Recurrent infections	(1) Perioperative antibiotics
	Poor nutrition	(1) Emphasis on adequate oral diet
		(2) Nutritional supplements
	Poor bone quality	(1) Bisphosphonate treatment

Major surgical insult	Increased intraoperative blood loss	(1) Hypotensive anaesthesia
		(2) Local haemostats used
		(3) Meticulous, sequential spinal exposure
		(4) Use of less implants
		(5) Use of cell saver
		(6) Use of allograft (no need for harvesting autologous bone from other sites)
	Reducing surgical time	(1) Use of single rod construct
	Pain effect on	(1) Limited use of IV opioids
	(i) chest,	(2) Aggressive chest physiotherapy
	(ii) mobilisation,	(3) Early postoperative mobilisation
	(iii) gastrointestinal system	(4) Supportive GI medication (including antiemetics and laxatives)

Scoliosis correction	Neurological	(1) Use of a single rod construct (less implant density)
	Infection	(1) Use of a single rod construct
		(2) Prophylactic antibiotics
	Respiratory compromise	(1) Thoracoplasty not performed
	Nonunion	(1) Extensive bone grafting
		(2) Postoperative support with spinal jacket
	Superior mesenteric artery syndrome	(1) Early instigation of oral diet
		(2) Nutritional supplements before and after surgery
		(3) Early postoperative mobilisation
